# The changing relationship between health burden and work disability of Australian cancer survivors, 2003–2017: evidence from a longitudinal survey

**DOI:** 10.1186/s12889-020-08710-9

**Published:** 2020-04-22

**Authors:** Rashidul Alam Mahumud, Khorshed Alam, Jeff Dunn, Jeff Gow

**Affiliations:** 1 Health Economics and Policy Research, Centre for Health Research, Faculty of Business, Education, Law and Arts, University of Southern Queensland, Toowoomba, Queensla nd-4350 Australia; 2 School of Commerce, University of Southern Queensland, Toowoomba, QLD-4350 Australia; 3 School of Social Sciences, Western Sydney University, Locked Bag 1797, Penrith, NSW 2751 Australia; 4 Health Economics Research, Health Systems and Population Studies Division, International Centre for Diarrhoeal Disease Research, Bangladesh (icddr,b), Dhaka, 1212 Bangladesh; 5 Department of Rajshahi, Health and Epidemiology Research, Rajshahi, 6205 Bangladesh; 6 Cancer Research Centre, Cancer Council Queensland, Fortitude Valley, Queensland, QLD 4006 Australia; 7 Prostate Cancer Foundation of Australia, St Leonards, NSW 2065 Australia; 8 School of Accounting, Economics and Finance, University of KwaZulu-Natal, Durban, 4000 South Africa

**Keywords:** Australia, Cancer survivors, Health burden, Longitudinal prospective study, Work disability

## Abstract

**Background:**

The purpose of this study was to examine the relationship between the cancer health burden and themagnitude of work disability on cancer survivors in Australia from 2003 to 2017.

**Methods:**

A longitudinal prospective study design was undertaken among cancer patients using data from the Household, Income and Labour Dynamics in Australia survey. The longitudinal effect was captured using a fixed effect multinomial logistic regression model, which predicted changes in the relationship between cancer burden and work disability level controlling for socio-demographic, lifestyle and life conditions predictors.

**Results:**

The prevalence of long-term disability among cancer survivors was 50%, with 18% of patients experiencing extreme work disability. The magnitude of disability levels increased significantly with the level of health burden. Cancer survivors who faced a severe health burden were at 5.32 times significantly higher risk of having work disability compared with patients who had no health burden. Other potential predictors, such as older patients (relative risk ratio, RRR = 1.82; 95% CI: 1.57, 5.87), those engaged in lower levels of physical activities (RRR = 1.91; 95% CI: 1.07, 3.40), those who drink alcohol (RRR = 1.29; 95% CI: 1.15, 1.49), and poor socioeconomic status (RRR = 1.28; 95% CI: 1.16, 2.23) were all significantly associated with extreme work disability.

**Conclusion:**

A substantial proportion of cancer survivors experienced work disability which was more pronounced with the magnitude of the cancer health burden. The different dimensions of disability might be prevented by introducing cancer survivor-specific evidence-based interventions, and incorporating comprehensive social support. Recommendations to improve public health policy aimed at reducing population-level unhealthy lifestyle behaviours include: using these findings to better outline the management of a sequelae course of treatment for cancer survivors; and identifying those who should undergo more intensive physical rehabilitation aimed at reducing their work disability level.

## Background

Worldwide, work participation of cancer survivors has seen a surge of attention in the last two of decades [1]. A cancer diagnosis can be a devastating and, often,life-threatening experience [2], which frequently results in short- or long-term disability [3–6] due to both health and economic burdens [7]. Cancer imposes a substantial burden in terms of reducing the autonomy of individuals to perform their general daily activities [1, 8]. Furthermore, a cancer diagnosis negatively affects employment status in terms of job opportunities, work participation and work ability due to the illness [1, 8]. The adverse side-effects of treatment results in physical and psychological limitations that can be a barrier to work participation [9]. However, the burden of physical disability levels varies by cancer stages and types [10]. Cancer survivors run a significantly high risk of unemployment and early retirement, and they have less opportunity to be re-employed [1]. A cohort study showed that 20% of cancer survivors reported disabilities due to cancer over a 5 year follow-up period [11]. An estimated 30% of cancer survivors reported work disabilities post-treatment [12]. However, a prospective cohort study confirmed that the employment opportunities of cancer survivors were adversely impacted by their recovery and health status [13]. Return to work participation may assist cancer survivors to recover faster, improve their quality of life, help return them to their former ‘normal’ life, increase their self-confidence, and may support them to overcome the negative side-effects of treatment [14, 15]. Furthermore, improvement of work participation of cancer survivors contributes to societal benefit, by reducing absenteeism, and reducing disability benefit payments and productivity losses [16]. Notably, cancer survivors’ earnings are 10% lower compared to non-cancer survivors [17]. Therefore, there is a greater need to provide supportive services (e.g., related to rehabilitation) to both help cancer survivors adapt to disability, and prevent work disability in this patient population.

In Australia, the incidence of cancer in individuals results in different disability levels for cancer survivors [2]. The long-term effects of cancer treatment are a significant cause of greater absenteeism, higher unemployment and early retirement [18], and overall reduced participation in work [2–6]. Approximately 40% of Australian cancer patients are of working age [19], with 46% being unable to return to employment after a cancer diagnosis [20], and 67% changing their employment status following diagnosed [21]. This results in a reduction of $1.7 billion to Australian gross domestic product (GDP) annually [20]. The impact of work disability constitute a substantial burden for people who have not had an occupation due to cancer, as well as to their families and employers. Furthermore, cancer-related treatment results in patients experiencing economic burden due to high out-of-pocket expenses (e.g., medicines and advanced treatments, including diagnostics), lost productivity, loss/reduction of household income, and other induced expenditure [22]. The majority of cancer patients depend on family, relatives and friends for physical and economic support during their course of treatment and in the last stages of the disease [23, 24]. Ultimately, cancer survivors are faced with a double burden in terms of their health and economic situation.

Existing studies have focused on cancer survivors’ characteristics and work participation, including in the United States [1, 10, 12, 16, 25–27], Canada [3, 13], South Korea [8], the Netherlands [5, 6, 9], and Belgium [4, 28]. A number of factors adversely influencing work participation of patients with cancer has been determined in different settings. These parameters are associated with patients’ socio-demographic characteristics (e.g., age, educational status and economic position) [5, 6, 9, 27], disease-related factors (e.g., tumor site, advanced tumor stage), advanced course of treatment (e.g., chemotherapy) [3, 6, 7, 27], and work-related factors (e.g., physical work demands) [1, 9]. The presence of comorbid conditions in cancer patients creates a higher likelihood of work-related disability [3]. That is, cancer survivors with poor health status were significantly correlated with a higher level of work disability [27]. A study conducted in the Netherlands found that cancer survivors who had experienced hormone therapy, metastatic disease, had limited physical strength, and limited workability, were strongly and adversely associated with a higher risk of work disability [5, 6]. The poor perceptions of cancer survivors, in terms of their health and work ability [6], their unhealthy behaviours (e.g., alcohol consumption), and their clinical stage [29] were also significant predictors in determining independent effects of their work disability levels.

In Australia, studies have been conducted among cancer patients exploring the psychological effects of current treatment or level of disability [30], association with work-related stress and cancer [31], and lost productivity due to cancer [20]. However, very limited evidence exists of the health burden in relation to work disability of cancer survivors in Australia. That is, potential factors associated with work disability of cancer survivors are poorly explored. This may be partially accounted for by various study designs, analytical rigour and follow-up periods. For instance, many international studies have used a limited number of predictors. The majority of the previous studies have been cross-sectional in nature, in terms of clinical and treatment perspectives. Thus, a comprehensive study is important to examine the impact of the health burden in relation to the magnitude of work disability as a long-term sequela of patients with cancer. There has been a recent surge of attention in the field of cancer survivorship, leading to efforts to identify and manage treatment-related sequelae, enhance quality of life, and improve the overall functioning of people who are receiving long-term follow-up care after cancer treatment.

Using longitudinal data from nationally representative Australian samples, these findings will help to improve the understanding of potential employment opportunities after a cancer diagnosis. In addition, these findings may be considered from different perspectives in cancer policy discussions: the cancer survivor (e.g., health status, work disability level, return to employment); the caregiver and the family (e.g., the health burden, reduction of socio-economic position, risk of poverty); the employer and co-workers (e.g., employment conditions, workload); the health care provider (e.g., supportive care needs, effective programs and interventions); and the community or society (e.g., economic and policy changes).

The present study aims to examine the health burden impact on the magnitude of work disability of cancer survivors after controlling several factors (e.g., socioeconomic, lifestyle, healthcare utilisation, and geographical location) over an extended period of 2003–2017. To achieve the research aim, the following three research questions (RQ) were posed:

RQ-1: What is the magnitude of work disability levels among cancer patients in Australia?

RQ-2: What is the longitudinal association between health burden and the magnitude of work disability among cancer patients in Australia over 2003–2017?

RQ-3: What are the potential predictors associated with the magnitude of work disability for cancer patients in Australia over this extended period?

## Methods

### Setting and data source

The study was conducted in the context of Australia. The Household, Income and Labour Dynamics in Australia (HILDA) study is a nationally representative household-based panel study. Data were collected from Australia residents aged 15 or over through face-to-face interviews and questionnaires, followed by re-interviews with the people in subsequent years. The details of the methods of data collection, including the sampling technique, have been reported elsewhere [32]. The overall goal of HILDA study is to collect data on the lives of Australian residents in terms of wealth, retirement, fertility, health, education, skills and abilities. Households and individuals are interviewed every year, allowing researchers to see how participants’lives change over time. Household longitudinal data, known as panel data, provides a more complete picture than cross-sectional data as it documents the life course each person takes. In many cases, panel data allows causal inferences that are more credible than those elicitedfrom other types of data. In particular, statistical methods known as ‘fixed-effects’ regression models can be employed to examine the effects of various factors on life outcomes such as long-term health conditions, earnings, unemployment, income and life satisfaction. These models can control for the effects of stable characteristics of individuals that are typically not observed, such as innate ability, motivation and optimism, that confound estimates of causal effects in cross-sectional settings.

### Study participants

**Fig. 1:**
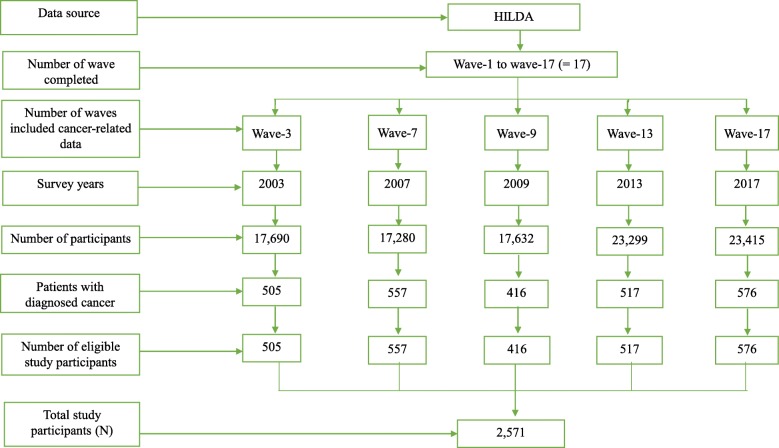
Distribution of study participants

The study was a sub-study with participants selected based on inclusion criteria of the HILDAsurvey [32], namely: 1) population aged 15 years or more (as per HILDA study participants), ii) diagnosed cancer patients, iii) longitudinal household members, and iv) willing to participate in HILDA study. The study participants were patients with cancer and data were derived from HILDA waves 3, 7, 9, 13 and 17, all of which had a health focus and asked specific questions related to cancer. Other survey waves were excluded from this study due to the paucity of cancer related information. A total of 2571 patients with diagnosed cancer were potential study participants (Fig. 1) from the five waves (505 patients from wave-3 in 2003, 557 patients from wave-7 in 2007, 416 patients from wave-9 in 2009, 517 patients from wave-13 in 2013 and 576 patients from wave-17 in 2017).

### Study design

The present study design was a mixed-longitudinal quantitative design in patients with cancer. Individuals who experienced a cancer diagnosis were examined with a focus on the magnitude of the cancer burden associated with their long-term-disability. To examine the mixed-longitudinal effects, this study hypothesised that several factors related to individuals’ socio-demographic characteristics, social factors, and disease-related symptomatic factors might influence outcome factors like disability. The combination of factors was expected to predict the patients’ long-term disability or adverse occurrence.

### Study variables

#### Outcome variable

Disability status and severity of disability were considered outcome measures. Work disability was measured by asking participants if they had any long-term health condition, impairment or disability that limited the kind or amount of work they could do. The magnitude of disability level was measured based on patients’ responses as “Could you pick a number between 0 and 10 to indicate how much your condition [s] limit [s] the amount of work you can do?” The severity of disability score was ranged from 0 to 10, with 10 indicating ‘able to do any work’ and 0 indicating ‘not at all’. The severity of disability level was defined as follows: (i) ‘no disability’ if disability score was equal to zero, (ii) ‘moderate disability’ for disability scores of 1 to 6, and (iii) ‘severe disability’ for disability scores of 7 to 10. The levels of disability were considered dependent variables in the analytical model.

### Explanatory variables

This study considered several demographic, socio-economic and health and lifestyle-related variables as predictors of long-term disability. The demographic variables included participant’s gender (male or female); age (< 25 years, 25–45 years, 46–65 years, or > 65 years), educational background (up to year 11, year 12, trade/certificate/diploma, or tertiary education), employment status (employed or unemployed), marital status (single, married, other including separated, divorced or widowed), and household size (< 3 members, 3 to 4 members, 5 or more members). Ethnic status was defined as Aboriginal or non-Aboriginal. Lifestyle factors include physical activity status (low, moderate, or high). Life condition-related variables such as satisfaction with household members, overall employment situation, financial situation and social supports were also considered as potential predictors. The level of satisfaction-related variables ranged from 0 (totally dissatisfied) to 10 (totally satisfied). Private insurance coverage of patients was dichotomous (insured or uninsured). Medication status was defined as ‘with medication’ or ‘without medication’.

To measure the impact upon quality of life the short form (SF)-36 was used. Health burden levels were defined as follows: (1) high burden if SF-36 score was less than 50, (2) moderate burden if SF-36 score was greater than or equal to 50 but less than 90, (3) no burden if SF-36 score was greater than or equal to 90 [33]. Remote locations were defined according to the accessibility to services and the Remoteness Index of Australia [34], and they were classified into five groups: major cities, inner regional, outer regional and remote or very remote. The index of relative socio-economic disadvantage (IRSD) was used to measure socio-economic status (SES). T This is a geographical area-based estimate of socio-economic status using a combination of income, education level and occupation where communities are ranked and categorised from economically disadvantaged to wealthy.he cut-off values for each of the quintiles are as follows: Q_1_ (IRSD ≤927.0), Q_2_ (927.0 > IRSD ≤965.8), Q_3_ (965.8 > IRSD ≤1001.8), Q_4_ (1001.8 > IRSD ≤1056.0), or Q_5_ (IRSD > 1056.0) [35].

### Statistical analysis

The overall cohort, and the subgroup that dropped out over the course of study, were characterized using frequency, means and proportions to summarise the participants’ characteristics in terms of demographics, unhealthy behaviours, life satisfaction, healthcare utilisation, remoteness and socioeconomic status. The association between the level of disability or disability status and the variables of greatest interest was analysed using the chi-square test or one way analysis of variance (ANOVA) where appropriate. During the analytical exploration, the present study also considered the missing data mechanisms as suggested by Rubin et al. (1976) and Little and Rubin (2002) [36, 37]. They classified the missing data process into three mechanisms: missing completely at random (MCAR), missing at random (MAR), and non-ignorable missing (NIM). In the study of work disability among cancer patients over time, missing data are closed to MCAR if the probability of attrition does not depend on the presence or severity of work disability (i.e., no disability, moderate disability or severe disability). A fixed-effects multinomial logistic regression model was used for analysis under the assumption of MCAR.

Both unadjusted and adjusted fixed-effect multinomial logistic regression models were used to identify the potential factors that had a significant role in the severity of disability level. In the regression model, the dependent variable (the severity of disability) was characterised by a categorical variable with three different levels (no disability, moderate disability or severe disability). The model was tested for sensitivity by the forward selection procedure (e.g., including and excluding specific variables) with the robust standard error. The predictor variables were included in the adjusted model only if any label of the predictor was significant at ≤5% risk level in the unadjusted regression model, which in turn was used to adjust for the effects of other potential confounders. Insignificant predictors were not included in the adjusted model. For independent variables, the category found to be least at risk of having an extreme or moderate disability level in the analysis was considered as the reference for constructing relative risk ratio (RRR), using fixed-effect multinomial logistic regression. During the data analysis, the study also looked at interaction effects in the analytical exploration, the interaction effects of the magnitude of long-term work disability in relation to RQ-3 by examining: age, employment status, life satisfactions, unhealthy behaviors and socio-economic status. We did not include the interaction effects in the results section and tables because the effects were insignificant in unadjusted model at a borderline risk level (*P* = 0.125). All data analyses were undertaken using the statistical software Stata/SE 13 (StataCorp, College Station, TX, USA). The statistical significance was considered at a 5% risk level.

## Results

### Description of study participants

Data from 2571 cancer patients were included in the analysis (Table 1). The percentage of male participants (54%) was higher than the female (46%). Approximately 45% of patients were senior, aged (> 65 years), followed by middle-aged (38%) (46 to 65 years). Approximately 47% had a middle or high school level education, with 15% of cancer patients having tertiary educationl qualifications. Approximately 45% of patients had limited exposure to physical activity, and only 23% of patients experienced high-level physical activities each week. Two-thirds of participants drank alcohol frequently. The majority of participants (90%) reported a moderate or high health burden in terms of their quality of life. In addition, 56% were insured, 72% received prescribed medication, and 60% lived in major cities.

**Table 1:** Characteristics of cancer patients by disability status

Variables	n (%) / n (mean)	Disability distribution among cancer survivors		*P*-value
		Any disability, n (%)	No disability, n (%)	
Sex				
*Male*	1398 (54.38)	711 (50.86)	687 (49.14)	0.353
*Female*	1173 (45.62)	575 (49.02)	598 (50.98)	
Age				
*< 25 years*	63 (2.45)	11 (17.46)	52 (82.54)	< 0.001
*25–45 years*	383 (14.90)	103 (26.89)	280 (73.11)	
*46–65 years*	975 (37.92)	409 (41.95)	566 (58.05)	
*> 65 years*	1150 (44.73)	763 (66.35)	387 (33.65)	
Educational attainment				
*Year 12 or below*	989 (38.47)	573 (57.94)	416 (42.06)	< 0.001
*Year 12*	220 (8.56)	97 (44.09)	123 (55.91)	
*Trade/certificate/diploma*	977 (38.00)	468 (47.90)	509 (52.10)	
*Tertiary*	385 (14.97)	148 (38.44)	237 (61.56)	
Employment status				
*Employed*	974 (37.88)	247 (25.36)	727 (74.64)	< 0.001
*Unemployed*	1597 (62.12)	1039 (65.06)	558 (34.94)	
Physical activity status				
*Low*	496 (45.38)	301 (60.69)	195 (39.31)	< 0.001
*Moderate*	346 (31.66)	176 (50.87)	170 (49.13)	
*High*	251 (22.96)	99 (39.44)	152 (60.56)	
Alcohol consumption (= yes)	1903 (74.02)	887 (46.61)	1016 (53.39)	< 0.001
Smoking exposure (= yes)	370 (14.39)	179 (48.38)	191 (51.62)	0.095
Health burden				
*No burden*	257 (10.00)	59 (22.96)	198 (77.04)	< 0.001
*Moderate burden*	1566 (60.91)	698 (44.57)	868 (55.43)	
*Severe burden*	748 (29.09)	529 (70.72)	219 (29.28)	
Private insurance coverage				
*Yes*	613 (56.08)	292 (47.63)	321 (52.37)	< 0.001
*No*	480 (43.92)	284 (59.17)	196 (40.83)	
Healthcare utilisation				
*Yes*	1093 (72.43)	682 (62.40)	411 (37.60)	< 0.001
*No*	416 (27.57)	115 (27.64)	301 (72.36)	
Life satisfaction with (mean scores)				
*Household members*	2571 (8.20)	8.23 (1.85)	8.17 (1.83)	0.786
*Employment*	2571 (3.37)	2.29 (3.62)	4.45 (3.98)	< 0.001
*Financial situation*	2571 (6.72)	6.54 (2.49)	6.89 (2.29)	0.005
*Social supports*	2571 (7.91)	7.79 (1.89)	8.03 (1.73)	< 0.001
Remoteness				
*Major cities*	1552 (60.37)	774 (49.87)	778 (50.13)	< 0.001
*Inner regional*	660 (25.67)	336 (50.91)	324 (49.09)	
*Outer regional*	314 (12.21)	158 (50.32)	156 (49.68)	
*Remote or very remote*	45 (1.75)	18 (40.00)	27 (60.00)	
Socioeconomic status				
*Q*_*1*_*(lowest 20%) (ref)*	516 (20.07)	293 (22.78)	223 (17.35)	< 0.001
*Q*_*2*_	595 (23.14)	322 (25.04)	273 (21.25)	
*Q*_*3*_	463 (18.01)	225 (17.50)	238 (18.52)	
*Q*_*4*_	534 (20.77)	238 (18.51)	296 (23.04)	
*Q*_*5*_*(highest 20%)*	463 (18.01)	208 (16.17)	255 (19.84)	
*Overall*	2571 (100.00)	1286 (50.02)	1285 (49.98)	

### Distribution of disability status among cancer patients (for RQ 1)

**Fig. 2:**
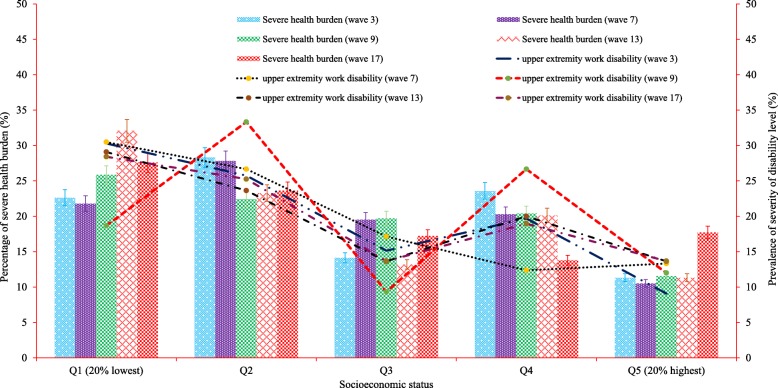
Unequal distribution of health burden and upper extremity work disability across socioeconomic status

Table 2 shows participants’ characteristics, overall and by disability status, across the selected variables. Half of the male patients experienced a long-term disability. The prevalence of disability increased significantly (*P < 0.001*) as patients aged (e.g., 17% for below 25 years, 27% for 25–45 years, 42% for 46–65 years, 66% for more than 65 years old). Approximately 58% of patients who had completed a middle or high school education level lived with a disability, followed by 48% of tertiary educated patients. The prevalence of disability was pronounced amongst the unemployed (65%), those who were poorly engaged in physical activities (61%) and those who were uninsured (59%). Furthermore, the proportion of disability was significantly aligned with the magnitude of cancer burden (e.g., 71% for severe burden, 45% for moderate burden and 23% for no burden). Regarding socio-economic status, the magnitude of work disability was found to be highest in the lowest socio-economic quintile. For example, patients who lived in the poorest households (23%) were significantly exposed to long-term disability (*P < 0.001*) compared with those in the richest households (16%). However, an upward trend in work disability levels was observed among the poorest cancer survivors during 2003–2017 (Fig. 2).

**Table 2:** Association of severity of disability and characteristics of cancer patients

Variables	Severity of disability						
	No disability		Moderate disability		Severe disability		*P*-value
	n	% (95% CI)	n	% (95% CI)	n	% (95% CI)	
Sex							
*Male*	816	58.37 (55.76, 60.93)	335	23.96 (21.80, 26.27)	247	17.67 (15.76, 19.76)	0.728
*Female*	672	57.29 (54.43, 60.10)	297	25.32 (22.91, 27.89)	204	17.39 (15.33, 19.67)	
Age							
*< 25 years*	46	73.02 (60.69, 82.58)	9	14.29 (7.56, 25.35)	8	12.70 (6.44, 23.51)	< 0.001
*25–45 years*	285	74.41 (69.80, 78.54)	61	16.00 (12.59, 19.95)	37	9.66 (7.08, 13.06)	
*46–65 years*	606	62.15 (59.06, 65.15)	214	21.95 (19.46, 24.66)	155	15.90 (13.73, 18.33)	
*> 65 years*	551	47.91 (45.03, 50.81)	348	30.26 (27.67, 32.98)	251	21.83 (19.53, 24.31)	
Educational attainment							
*Year 12 or below*	489	49.44 (46.33, 52.56)	284	28.72 (25.98, 31.62)	216	21.84 (19.37, 24.53)	< 0.001
*Year 12*	147	66.82 (60.31, 72.74)	36	16.36 (12.03, 21.87)	37	16.82 (12.42, 22.37)	
*Trade/certificate/diploma*	588	60.18 (57.08, 63.21)	234	23.95 (21.38, 26.73)	155	15.86 (13.70, 18.29)	
*Tertiary*	264	68.57 (63.75, 73.02)	78	20.26 (16.53, 24.58)	43	11.17 (8.38, 14.73)	
Employment status							
*Employed*	740	75.98 (73.19, 78.56)	158	16.22 (14.04, 18.67)	76	7.80 (6.27, 9.66)	< 0.001
*Unemployed*	748	46.84 (44.40, 49.29)	474	29.68 (27.49, 31.97)	375	23.48 (21.47, 25.63)	
Physical activity status							
*Low*	233	46.98 (42.61, 51.39)	126	25.40 (21.76, 29.43)	137	27.62 (23.86, 31.73)	< 0.001
*Moderate*	197	56.94 (51.65, 62.07)	101	29.19 (24.63, 34.21)	48	13.87 (10.61, 17.94)	
*High*	176	70.12 (64.15, 75.47)	55	21.91 (17.21, 27.47)	20	7.97 (5.19, 12.04)	
Alcohol consumption (= yes)	1175	61.74 (59.54, 63.90)	448	23.54 (21.69, 25.50)	280	14.71 (13.19, 16.38)	< 0.001
Smoking exposure (= yes)	224	60.54 (55.46, 65.41)	82	22.16 (18.21, 26.69)	64	17.30 (13.77, 21.50)	0.455
Health burden							
*No burden*	248	96.50 (93.40, 98.17)	7	2.72 (1.30, 5.61)	2	0.78 (0.19, 3.07)	< 0.001
*Moderate burden*	1018	65.01 (62.61, 67.33)	403	25.73 (23.63, 27.96)	145	9.26 (7.92, 10.80)	
*Severe burden*	222	29.68 (26.51, 33.06)	222	29.68 (26.51, 33.06)	304	40.64 (37.17, 44.21)	
Private insurance coverage (= yes)	362	59.05 (55.10, 62.89)	155	25.29 (22.00, 28.89)	96	15.66 (12.99, 18.76)	0.005
Healthcare utilisation (= yes)	495	45.29 (42.35, 48.26)	340	31.00 (28.43, 33.92)	258	23.60 (21.18, 26.22)	< 0.001
Life satisfaction with (mean scores)							
*Household members*	1488	8.18 (8.09, 8.27)	632	8.24 (8.10, 8.38)	451	8.20 (8.01, 8.39)	0.034
*Employment*	1488	4.30 (4.10, 4.51)	632	2.48 (2.20, 2.77)	451	1.53 (1.25, 1.82)	< 0.001
*Financial situation*	1488	6.94 (6.83, 7.06)	632	6.59 (6.40, 6.78)	451	6.15 (5.91, 6.39)	< 0.001
*Social supports*	1488	8.01 (7.93, 8.09)	632	7.92 (7.78, 8.06)	451	7.61 (7.42, 7.80)	< 0.001
Remoteness							
*Major cities*	945	60.89 (58.43, 63.29)	347	22.36 (20.35, 24.50)	260	16.75 (14.97, 18.7)	< 0.001
*Inner regional*	344	52.12 (48.30, 55.92)	194	29.39 (26.04, 32.99)	122	18.00 (15.70, 21.64)	
*Outer regional*	169	53.82 (48.27, 59.28)	80	25.00 (20.95, 30.60)	65	20.70 (16.57, 25.55)	
*Remote or very remote*	30	66.67 (51.65, 78.92)	11	24.44 (13.99, 39.16)	4	8.89 (3.34, 21.61)	
Socioeconomic status							
*Q*_*1*_*(lowest 20%) (ref)*	258	50.00 (45.69, 54.31)	133	25.78 (22.18, 29.73)	125	24.22 (20.72, 28.12)	< 0.001
*Q*_*2*_	312	52.44 (48.41, 56.43)	163	27.39 (23.96, 31.13)	120	20.17 (17.13, 23.59)	
*Q*_*3*_	280	60.48 (55.94, 64.84)	120	25.92 (22.12, 30.11)	63	13.61 (10.77, 17.05)	
*Q*_*4*_	329	61.61 (57.41, 65.65)	119	22.28 (18.95, 26.02)	86	16.10 (13.22, 19.48)	
*Q*_*5*_*(highest 20%)*	309	66.74 (62.31, 70.89)	97	20.95 (17.48, 24.90)	57	12.31 (9.61, 15.63)	
*Overall*	1488	57.88 (55.96, 59.77)	632	24.58 (22.95, 26.29)	451	17.54 (16.12, 19.06)	

*P*-value was derived using chi-square test or one way analysis of variance (ANOVA) where appropriate

### Association between severity of disability and patient’s characteristics (for RQ 2)

The age distribution of patients contributed significantly (*P < 0.001*) to the magnitude of long-term disability (Table 2). Educational background was significantly associated with disability level (*P < 0.001*). Several patient characteristics including employment status (*P < 0.001*), insurance coverage (*P = 0.005*), utilisation of prescribed medication (*P < 0.001*), life satisfaction related-factors (*P < 0.05*), alcohol consumption (*P < 0.001*), geographical location (*P < 0.001*) and socio-economic status (*P < 0.001*) were significant predictors of disability level. Furthermore, the level of physical activity (*P < 0.001*) and the health burden related to cancer (*P < 0.001*) were dominant variables for the severity of the disability.

### Factors influencing disability among patients with cancer (for RQ 3)

Table 3 shows the results of the fixed effect multinomial logistic regression analysis. In the final model, age, educational achievement, physical activities, health burden associated with cancer, utilisation of prescribed medication, patients living in a regional location, and those in the poorest households were significant predictors of a higher risk of long-term disability. An aged patient (> 65 years old) was at 1.82 times higher risk of having an extreme disability (RRR = 1.82; 95% CI: 1.57, 5.87) compared with a younger patient (< 25 years), while being 1.40 times more likely to have a moderate level of disability (RRR = 1.40; 95% CI: 1.09, 4.00). Patients who were unemployed had a significantly higher risk of being affected by severe disability (RRR = 2.01; 95% CI: 1.15, 3.50) or a moderate level of disability (RRR = 1.55; 95% CI: 1.01, 2.39) compared with their employed counterparts. Similarly, patients who performed a lower level of physical activities were 1.91 times more likely to have an extreme disability (RRR = 1.91; 95% CI: 1.07, 3.40) compared with patients engage in high-level physical activities. Patients who had an extreme health burden associated with cancer were at approximately five times significantly higher risk of experiencing a severe or moderate disability level compared with patients who reported excellent health status. Unhealthy behavioural factors like alcohol consumption (RRR = 1.29; 95% CI: 1.15, 1.49) were associated with work disability compared with patients who had not consumed alcohol. The risks of having an extreme disability (RRR = 1.28 times) or moderate disability level (RRR = 1.36 times) of cancer patients who lived in the poorest households were more pronounced compared with their richer counterparts.

**Table 3:** Factors influencing severity of disability of cancer patients

Variables	Moderate disability vs No disability		Severe disability vs No disability	
	Un-adjusted model	Adjusted model	Un-adjusted model	Adjusted model
	RRR (95% CI)	RRR (95% CI)	RRR (95% CI)	RRR (95% CI)
Health burden				
*No burden (= ref)*	1.00	1.00	1.00	1.00
*Moderate burden*	7.88*** (4.26, 10.04)	4.03*** (3.56, 9.99)	5.66*** (4.35, 7.79)	5.92** (1.38, 25.40)
*Severe burden*	7.27*** (6.28, 18.4)	5.43*** (3.34, 7.81)	6.80*** (4.78, 9.09)	5.32*** (2.75, 11.60)
Female (ref = male)	1.08 (0.89, 1.30)		1.00 (0.81, 1.24)	
Age				
< 25 years (= ref)	1.00	1.00	1.00	1.00
25–45 years	1.09 (0.51, 2.35)	1.12 (0.37, 3.37)	0.75 (0.33, 1.70)	0.85 (0.24, 2.97)
46–65 years	1.80 (0.87, 3.75)	1.31 (0.47, 3.66)	1.47 (0.68, 3.18)	1.39 (0.45, 4.34)
> 65 years	3.23*** (1.56, 6.68)	1.40** (1.09, 4.00)	2.62*** (1.22, 5.63)	1.82*** (1.57, 5.87)
Educational achievement				
Year 11 or below (= ref)	1.00	1.00	1.00	1.00
Year 12	0.42*** (0.28, 0.62)	1.36 (0.82, 2.24)	0.57*** (0.38, 0.85)	0.77 (0.43, 1.39)
Trade/certificate/diploma	0.69*** (0.56, 0.85)	1.10 (0.51, 2.36)	0.60*** (0.47, 0.76)	1.54 (0.68, 3.47)
Tertiary or university	0.51*** (0.38, 0.68)	1.00 (0.62, 1.61)	0.37*** (0.26, 0.53)	0.77 (0.43, 1.36)
Unemployed (ref = employed)	2.97*** (3.65, 0.62)	1.55** (1.01, 2.39)	4.88*** (6.37, 0.85)	2.01*** (1.15, 3.50)
Marital status				
Single (= ref)	1.00	1.00	1.00	1.00
Married	1.68*** (1.22, 2.32)	1.41 (0.79, 2.54)	1.33 (0.94, 1.88)	0.69 (0.37, 1.31)
Others	2.25*** (1.60, 3.16)	1.44 (0.79, 2.62)	1.86*** (1.29, 2.68)	0.89 (0.47, 1.69)
Physical activity status				
Low	1.73*** (1.19, 2.51)	0.98 (0.64, 1.50)	5.17*** (3.11, 8.60)	1.91** (1.07, 3.40)
Moderate	1.64*** (1.11, 2.41)	1.30 (0.85, 2.00)	2.14*** (1.22, 3.75)	1.43 (0.77, 2.65)
High (= ref)	1.00	1.00	1.00	1.00
Alcohol consumption (ref = no)	1.54*** (1.25, 1.91)	0.94 (0.65, 1.35)	2.29*** (1.83, 2.88)	1.29*** (1.15, 1.49)
Smoking exposure (ref = no)	1.19 (0.91, 1.56)		1.07 (0.79, 1.45)	
Private insurance coverage (ref = yes)	1.22 (0.91, 1.62)	0.88 (0.62, 1.24)	1.68*** (1.22, 2.32)	1.02 (0.68, 1.53)
Healthcare utilisation (ref = yes)	4.62*** (3.34, 6.39)	0.29*** (0.19, 0.44)	8.13*** (5.15, 12.83)	0.34*** (0.20, 0.60)
Life satisfaction with				
*Household members*	1.02 (0.97, 1.07)	1.00 (0.90, 1.10)	1.01 (0.95, 1.07)	1.01 (0.90, 1.13)
*Employment*	0.89*** (0.87, 0.91)	0.99 (0.95, 1.04)	0.81*** (0.79, 0.84)	0.96 (0.91, 1.02)
*Financial situation*	0.94*** (0.90, 0.98)	0.95 (0.88, 1.03)	0.88*** (0.84, 0.91)	0.94 (0.86, 1.02)
*Social supports*	0.97 (0.92, 1.02)	1.00 (0.90, 1.10)	0.89*** (0.84, 0.94)	0.98 (0.88, 1.10)
Remoteness				
*Major cities (= ref)*	1.00	1.00	1.00	1.00
*Inner regional*	1.54*** (1.24, 1.90)	1.75*** (1.21, 2.52)	1.29* (1.01, 1.65)	1.60** (1.04, 2.48)
*Outer regional*	1.29 (0.96, 1.73)	0.95 (0.55, 1.63)	1.40* (1.02, 1.92)	1.27 (0.70, 2.31)
*Remote or very remote*	1.00 (0.50, 2.01)	0.80 (0.26, 2.43)	0.48 (0.17, 1.39)	0.22 (0.03, 1.81)
Socioeconomic status				
*Q*_*1*_*(lowest 20%)*	1.64*** (1.21, 2.24)	1.36** (1.09, 2.33)	2.63*** (1.84, 3.74)	1.28*** (1.16, 2.23)
*Q*_*2*_	1.66*** (1.24, 2.24)	1.44 (0.87, 2.39)	2.09*** (1.47, 2.97)	1.25 (0.68, 2.32)
*Q*_*3*_	1.37* (1.00, 1.87)	1.49 (0.87, 2.54)	1.22 (0.82, 1.81)	1.09 (0.56, 2.14)
*Q*_*4*_	1.15 (0.85, 1.57)	1.21 (0.72, 2.01)	1.42 (0.98, 2.05)	1.36 (0.73, 2.52)
*Q*_*5*_*(highest 20%) (= ref)*	1.00	1.00	1.00	1.00

*RRR* Relative risk ratio, *CI* Confidence interval, *ref*. Reference group

## Discussion

Cancer is significantly correlated with workdays lost and high levels of work-related disability [29, 38–40]. The main objectives of this study were to investigate the magnitude of work disability due to a cancer diagnosis and measure the longitudinal association between health burden and disability, and the potential predictors of work disability of cancer patients. The study results show that 50% of cancer patients experienced a long-term disability, whereas approximately 18% of patients had reached an extreme level of work disability. Furthermore, the prevalence of disability was pronounced in relation to the level of the cancer burden (e.g., 71% for severe burden, 45% for moderate burden, and 23% for no burden), aged patients (66%), and unemployed patients (65%), those engaged in limited physical activities (61%), the uninsured (59%), and the poorest socio-economic group (23%). Potential predictors, which included factors such as age, those who exercise less or not at all, those who have an extreme health burden, and engage in unhealthy behaviours (e.g., alcohol consumption), were significantly associated with a higher risk of having an extreme disability.

The results showed that a higher risk of a severe or moderate disability level was pronounced among cancer patients who faced an extreme health burden, compared with patients who reported an excellent health status. A previous study found that poor health status of cancer patients resulted in greater functional disability (e.g., specific task difficulties) [41, 42]. However, the prevalence of long-term disability was more pronounced in combination with a cancer diagnosis [5, 6, 12, 27, 29]. Advanced cancer treatments can damage healthy cells or organs [43]. For example, radiation and chemotherapy may impose short and long-term health problems and impact on the spinal cord, nerves and brain, which then may significantly contribute to long-term adverse outcomes like work-related disability. In the context of Australia, a significant proportion (46%) of cancer patients are unable to return to employment after their diagnosis [20].

Furthermore, work disability leads to a substantial economic burden on society, individuals and their families, resulting in a reduction of $1.7 billion annually to GDP in Australia [20] and an approximately 5% GDP reduction in the Organisation for Economic Co-operation and Development (OECD) countries [44]. Therefore, cancer survivors may require psycho-social healthcare services and other therapeutic modalities, such as physical and occupational therapy, to assist in their return to a productive work life. Cancer patients with physically demanding jobs may require assistance during treatment, and possibly physical rehabilitation following treatment, in order to minimize morbidity. However, developing new and improved treatments with fewer side effects is another potentially important strategy to reduce cancer-related disability.

The results indicate that elderly cancer patients (older than 65 years) were at a significantly higher risk of having an extreme disability compared with younger patients (< 25 years). This finding is consistent with a previous study, which revealed that elderly cancer patients reported significantly more functional disabilities [45], required more assistance with daily living activities [46], and had deficits in performing work-related activities in terms of their physical ability [41, 47]. Thus, several factors might influence the reduction in their physical functioning. For example, a course of advanced cancer treatment is associated with considerable physical and psychological side effects in elderly cancer patients (e.g., weight change, muscle loss, fatigue and physical weakness) [48], and having multiple comorbidities [3, 27, 29] will presumably contribute to reduced daily activities. Moreover, an elderly cancer patient may have a limited acceptance of advanced treatment and health outcomes that may then contribute to a greater burden of health [48]. This result indicates that rehabilitation-related interventions (e.g., occupational and physical therapies) are essential to prevent ongoing work disability of cancer patients [49], and is an emerging cancer research area, particularly focused on the elderly [50].

The study results found that low level or no physical activities in cancer patients was strongly associated with an extreme level of work-related disability compared with patients engaged in high-level physical activity. This finding is consistent with other research [38, 51–54], whereby authors found that limited physical activity levels were significantly associated with a higher risk of work disability among cancer patients. Further, a number of previous studies have proven that physical activity plays an effective role in ensuring improved health status [55], reducing the risk of developing future cancers [54], and also expressively contributing to lower mortality risk [56], which ultimately produces significant health benefits and reduces medical expenditures and treatment outcome disparities [55]. In terms of cancer risk, high levels of physical activities (compared with low levels) played a significant role in prevention of several cancers (e.g., 42% for gastrointestinal cancer, 23% for renal cancer, and 20% for myeloid leukemia) [57]. This included averting genetic damage, improving the immune system, reducing chronic infections, and controlling cancer cells [57]. Several hypotheses and mechanisms have been suggested regarding the anti-cancer effects of physical activities. The American Cancer Society guidelines for cancer survivors [58] recommend daily physical activities, including a continuation of normal daily life activities immediately after diagnosis, which help to significantly reduce physical stamina and muscle strength erosion as well as anxiety levels, thereby resulting in the prevention of long-term adverse health outcomes (e.g., work-related disability) [59].

This study results found an increased risk of work disability among cancer patients who consumed alcohol compared with patients who did not. In this study, alcohol consumption had a robust effect on patient outcomes. Formal drinkers represented two-thirds (≈ 75%) of the cohort and had a 46% greater risk of disability. The last Global Burden of Disease study, conducted in 2016, found a similar result, namely that alcohol consumption was a dominating determinant for higher risk of having a disability [60]. The World Health Organisation (WHO) has suggested that harmful alcohol consumption causes a high burden of disease, including cancer [61], which is often underappreciated [60]. This finding has further implications for the reform of public health policy, and decreasing population-level alcohol consumption should be recommended.

The risks of having an extreme disability level amongst cancer patients who lived in the poorest households were more pronounced compared with their richer counterparts. Recent studies have confirmed this result with disadvantaged socio-economic status of cancer survivors being negatively associated with long-term health effects or work-related disability [62, 63]. Some studies have also provided evidence that the magnitude of the cancer burden is negatively associated with socio-economic status [16, 31–34]. Furthermore, adverse health outcomes (e.g., worse health status, long and short-term disability and shorter life expectancy) were disproportionately found in poorer people as opposed to those with higher socio-economic status [13, 16, 31, 33, 64–71]. Contribtuing factors to the high rates of long term health impacts among the poorest groups includes higher tobacco rates [16, 27], economic burden [35, 36], increased mental illness [72], lack of health education and awareness [73], and less access to competent and effective health care services [73].

Low productivity, loss/reduction of household income, and increased healthcare expenditure are pronounced amongst the poorest cancer patients. Growing socio-economic inequalities of cancer outcomes need the attention of governments, health systems and decision makers. For example, Cancer Australia has an optimal care pathway project, which has already addressed several cancer types. Such initiatives might help to reduce socio-economic inequalities, which are related to poverty, gender, education, and health, and should promote universal access to health care which can further enhance both socio-economic and human development.

The ability to continue in the labour force, and allowing an individual the choice to do so, signifies a key aspect of the health status often threatened by disease. Long-term disability threatens the economic well-being of survivors and their families. Additionally, the health status of cancer patients who are restricted in their capacity to work may be affected by the loss of identity, life satisfaction, and social relationships that work often provides. Cancer survivorship, work disability and employment may be considered from different perspectives: the cancer survivor (e.g., health status, work disability level and return to employment), the caregiver and the family (e.g., the health burden, reduction of socioeconomic position and risk for poverty), the employer and co-workers (e.g., employment conditions and workload), the health care provider (e.g., supportive care needs, effective programs and interventions), and the community or society (e.g., economic and policy changes).

This study includes some caveats. Study participants were accessed from the HILDA survey, which covers health, economic, employment, income and health characteristics of household members aged 15 years and older. Children who suffered from cancer were excluded from this study. Examining the long-term work disability is widely perceived to have substantial potential as an endpoint in health outcomes research; however, results are partially dependent upon study methods and outcome variables of interest. The participants of the present study were derived from the protocol “HILDA study” [32], wherein long-terms health conditions of cancer patients might change for independent study designs as well as application of survey instruments.

This study findings established a relationship between overall cancer burden and work-related disability among cancer survivors, which might vary in terms of cancer stages and types of cancer. The authors were not able to estimate the cancer-specific health burden nor the work disability of cancer survivors due to the paucity of relevant data. Further, the study findings were based on self-reported responses that might have been impacted by respondents’ prejudice (e.g., silence and over-response), and by problems in understanding and interpreting the survey questions.

Despite these limitations, this study has noteworthy strengths including the use of a prospective design of long term follow-ups, and the application of well-validated and reliable longitudinal wave measures of the impacts of cancer diagnosis on the health burden and work disability of individuals over the 2003–2017 period. The study population was ethnically, geographically, and socio-economically diverse. Furthermore, this study included several potential confounding analytical factors that were not present in previous studies. For this study, data were gathered from five-waves of the HILDA survey of cancer survivors. The length of the survey period may have introduced uncontrolled bias, as changes in health status are not instantaneous and might emerge only after time, which was not captured in this study. Due to funding restrictions, the authors were unable to consider cancer patients who registered for cancer surveillance as well as received health care from health facilities (e.g., private clinics, community clinics, secondary or tertiary hospitals). Due to the paucity of cancer-related data in HILDA study, the authors were unable to perform cancer-specific analysis and period of treatment analysis. Future research is required using a similar study design, perspective and analytical methods in terms of cancer-specific exploration.

## Conclusions

This study has identified a high rate of work-related disability that leads to a substantial decrease in a cancer survivor’s socio-economic position. Several demographic, social, lifestyle and health burden variables were associated with the magnitude of disability. The findings have further implications for improving public health policy, and reducing population-level unhealthy lifestyle behaviours which should be recommended. The study results could be used to better outline the management of a sequelae course of treatment for those who should undergo more intensive physical rehabilitation aimed at reducing work disability levels. This may apply to cancer survivors who choose or need to work after cancer diagnosis and treatment especially those still active in the work force. This is important in light of the increasing prevalence of cancer and fortunately, the growing numbers of patients surviving cancer in Australia, and the likelihood of the development of impairments and activity limitations after cancer treatment. It is also significant for health care providers, including physical and occupational therapists and oncologists, who should be aware of the unique problems that challenge this population and who should advocate for prevention and evidence-based interventions. Therefore, it is recommended that effective and efficient cancer survivor-specific evidence-based interventions be developed to reduce the impacts of work disability by incorporating comprehensive social supports which ultimately, have the potential to affect the trajectory of the cancer burden in a positive way.

## Data Availability

The Household, Income and Labour Dynamics in Australia (HILDA) data are used under strict licensing. Data can be potentially obtained subject to a peer-reviewed application. Further details are available at: https://www.melbourneinstitute.com/hilda/

## Abbreviations

IRSD: The index of relative socio-economic disadvantage
SES: Socioeconomic status,
SF-36: Short form
CI: Confidence Interval
HILDA: Household, Income and Labour Dynamics in Australia
